# Evaluation of a tailored, multi-component intervention for implementation of evidence-based clinical practice guidelines in primary care physical therapy: a non-randomized controlled trial

**DOI:** 10.1186/1472-6963-14-105

**Published:** 2014-03-04

**Authors:** Susanne Bernhardsson, Maria EH Larsson, Robert Eggertsen, Monika Fagevik Olsén, Kajsa Johansson, Per Nilsen, Lena Nordeman, Maurits van Tulder, Birgitta Öberg

**Affiliations:** 1 Närhälsan Öckerö Rehabilitation, Region Västra Götaland, Hönö, Sweden; 2 Department of Medical and Health Sciences, Division of Physiotherapy, Linköping University, Linköping, Sweden; 3 Närhälsan, Research and Development Primary Health Care, Region Västra Götaland, Gothenburg, Sweden; 4 The Sahlgrenska Academy, Institute of Neuroscience and Physiology/Physiotherapy, University of Gothenburg, Gothenburg, Sweden; 5 Närhälsan Mölnlycke Health Care Centre, Mölnlycke, Sweden; 6 The Sahlgrenska Academy, Institute of Medicine/Department of Public Health and Community Medicine/Primary Health Care, University of Gothenburg, Gothenburg, Sweden; 7 Department of physical therapy and occupational therapy, Sahlgrenska University Hospital, Gothenburg, Sweden; 8 Department of Medical and Health Sciences, Division of Health Care Analysis, Linköping University, Linköping, Sweden; 9 Närhälsan, Research and Development Primary Health Care, Region Västra Götaland, Borås, Sweden; 10 Department of Health Sciences & EMGO + Institute for Health and Care Research, VU University, Amsterdam, The Netherlands

**Keywords:** Implementation, Physical therapy, Evidence-based practice, Practice guidelines

## Abstract

**Background:**

Clinical practice guidelines are important for transmitting research findings into practice and facilitating the application of evidence-based practice (EBP). There is a paucity of knowledge about the impact of guideline implementation strategies in primary care physical therapy. The aim of this study was to evaluate the effect of a guideline implementation intervention in primary care physical therapy in western Sweden.

**Methods:**

An implementation strategy based on theory and current evidence was developed. A tailored, multi-component implementation intervention, addressing earlier identified determinants, was carried out in three areas comprising 28 physical therapy practices including 277 physical therapists (PTs) (intervention group). In two adjacent areas, 171 PTs at 32 practices received no intervention (control group). The core component of the intervention was an implementation seminar with group discussions. Among other components were a website and email reminders. Data were collected at baseline and follow-up with a web-based questionnaire. Primary outcomes were the self-reported awareness of, knowledge of, access to, and use of guidelines. Secondary outcomes were self-reported attitudes toward EBP and guidelines. Analyses were performed using Pearson’s χ^2^ test and approximative z-test.

**Results:**

168 PTs (60.6%) in the intervention group and 88 PTs (51.5%) in the control group responded to the follow-up questionnaire. 186/277 PTs (67.1%) participated in the implementation seminars, of which 97 (52.2%) responded. The proportions of PTs reporting awareness of (absolute difference in change 20.6%, p = 0.023), knowledge where to find (20.4%, p = 0.007), access to (21.7%, p < 0.001), and frequent use of (9.5%, NS) guidelines increased more in the intervention group than in the control group. The proportion of PTs reporting frequent guideline use after participation in the implementation seminar was 15.2% (p = 0.043) higher than the proportion in the control group. A higher proportion considered EBP helpful in decision making (p = 0.018). There were no other significant differences in secondary outcomes.

**Conclusions:**

A tailored, theory- and evidence-informed, multi-component intervention for the implementation of clinical practice guidelines had a modest, positive effect on awareness of, knowledge of, access to, and use of guidelines, among PTs in primary care in western Sweden. In general, attitudes to EBP and guidelines were not affected.

## Background

The concept of evidence-based practice (EBP) [1] is increasingly permeating physical therapy practice, although there is considerable variation in the extent to which EBP is actually applied [2-5]. Physical therapists (PTs) use treatment methods with strong or moderate evidence of effect, but also methods with limited or no effect [6-9]. The body of evidence for physical therapy treatment is growing at a fast pace [10], making it challenging for clinicians to keep up with the latest findings. To bridge the gap between research and practice and facilitate the uptake of research findings in physical therapy practice, evidence-based clinical practice guidelines are increasingly being produced.

The use of physical therapy guidelines has been shown to contribute to EBP, improve the quality of care, and decrease costs [2,8,11]. However, the availability and use of guidelines in different countries and settings also tend to vary [3,5,12-14]. In Sweden, few guidelines for physical therapy treatments are available and less than half of the PTs in a recent survey stated that they use guidelines on a regular basis [5].

Evidence-based guidelines need to be accompanied by evidence-based implementation [15]. There is growing evidence that active, multi-component strategies are effective in implementing change in professional behavior [16-21], and that components such as printed educational material (*e.g.,* guidelines) [22], interactive education [23] and reminders [24] may be beneficial. Tailored implementation targeting specific barriers and facilitators has received increased attention and evidence for this approach is also growing [21,25-27]. A recent Cochrane review concluded that a tailored implementation intervention is more likely to improve professional practice than no intervention or dissemination of guidelines [25]. Different approaches have been suggested to link determinants with strategies, from theory-based [28] to more pragmatic “common sense” approaches [29,30].

Implementation of guidelines or other measures to change practice behavior needs to be supported by theories or models [17,31-33]. Theory can facilitate the implementation and increase the possibility to draw general conclusions on the effectiveness of the implementation strategy [32]. Using theory provides a process and structure to support the development of a strategy or intervention and to guide its evaluation, thereby facilitating a better understanding of the generalizability and replicability of implementation interventions [34]. There is some evidence that behavior change interventions that are informed by theory are more effective than those that are not [35]. However, few guideline implementation interventions have so far been based on theory [33].

In physical therapy, relatively little is known about the effectiveness of various implementation strategies. A tailored, active, multi-component implementation strategy has been recommended [36]. No study on a tailored strategy has been found, and there is only tentative evidence that active, multifaceted strategies are more effective than passive, single intervention strategies [37-39]. A systematic review on implementation of guidelines in physical therapy concluded that an active, multifaceted strategy was effective in improving knowledge and behavior, but not attitudes, patient outcomes, or cost of care and that the effects were mostly small [39]. Hence, there is inconclusive evidence as to which strategies are most likely to be effective in different contexts. Studies investigating the implementation of guidelines in primary care physical therapy are scarce [40,41] and none have been performed in Sweden.

A project to develop and implement evidence-based clinical practice guidelines for primary care physical therapy was initiated by a regional health authority in western Sweden. To introduce the new guidelines, a tailored, multi-component implementation intervention was developed, combining a theory-informed, evidence-based strategy with a pragmatic approach. The aim of this study was to evaluate the effectiveness on process outcomes of this guideline implementation intervention compared with no intervention.

## Methods

### Design and setting

The design of this study was two-fold and comprised a systematic implementation strategy development component and a non-randomized controlled trial in which the effectiveness of the implementation intervention was evaluated in comparison with no intervention. Data were collected at baseline and follow-up. The study took place within the county council Region Västra Götaland in western Sweden, providing first-line primary care of both acute and long-term nature to 1.6 million inhabitants. At the physical therapy practices, PTs primarily treat patients with musculoskeletal disorders; the most common complaints are low back pain, neck pain, and subacromial pain. There are 60 physical therapy practices in five areas within the county council, which were allocated to intervention (three areas, 28 practices) or control group (two adjacent areas, 32 practices) based on geographic location. The study was conducted between November 2010 and November 2011.

### Participants

**Figure 1:**
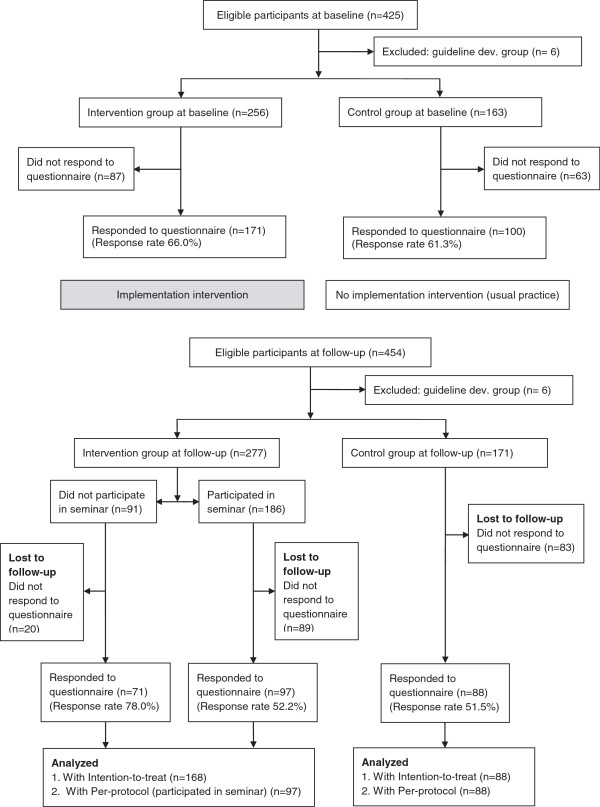
Flow chart of participants through the study.

All PTs employed at the 60 physical therapy practices were eligible to participate in the study. At the time of the baseline data collection, 425 PTs were employed in primary care by the authority and at follow-up, 454 PTs were employed. The guideline project team members, who were also employed by the county council, were excluded (n = 6). Figure 1 illustrates participant flow through the study.

### Guideline development

**Figure 2:**
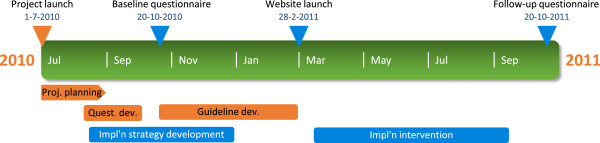
**Project timeline.** Guideline development, guideline implementation strategy development, guideline implementation intervention, and evaluation time points. Blue elements are part of this study.

The initiative to the guideline development and implementation was taken by PT managers within the county council, who perceived a variation in physical therapy practice and therefore a need for evidence-based practice guidelines. An integrated organizational development/research project was planned. Support from senior management of the county council, as well as funding of the project, was secured. Guideline development, needs analysis, and implementation strategy development were carried out in parallel processes within the integrated project (Figure 2). The guideline development was performed by a project team of six primary care PTs, in a systematic process guided by a 7-step guideline development model by Grol et al. [17]. The development process included systematic database searches, critical appraisal of the evidence base using GRADE [42], and formulating evidence-based practice recommendations. The guideline format followed recommendations from AGREE II [43]. The guidelines consisted of a brief summary on the first page; a brief introduction to the topic with up-to-date data on definition of the condition, prevalence and prognosis; recommendations on patient management according to strength of evidence; and a detailed reference list. Detailed search strategy and search results, summaries of reference articles, recommended outcome measures, and patient information leaflets were provided in appendices to the guidelines.

### Implementation strategy development

**Figure 3:**
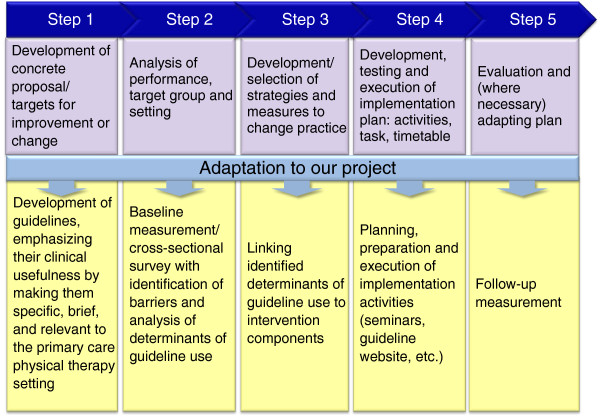
**Adaptation of Grol’s et al. 5-step model for planning and executing an implementation process**[17]**.**

**Figure 4:**
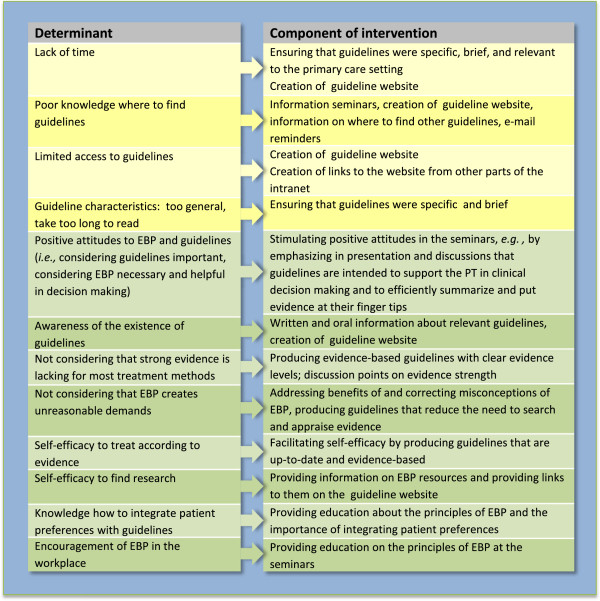
**Matching determinants to intervention components.** Most important barriers (yellow) and facilitators (green) of guideline use identified in previous cross-sectional survey [5].

An implementation strategy was developed based on prevailing implementation theory [17], barriers and facilitators identified in the target population [5], and evidence from previous research. The latter included strategies that are theory-based [31,44], active [38], multi-faceted [39], and tailored [25]. The 5-step model for implementation by Grol et al. [17] was selected as a framework for the implementation, because it is based on a comprehensive overview of theories for behavioral change, integrates several theories that we considered relevant, and provides a structured approach for the implementation process. The model, and how it was adapted for the specific clinical and organizational context and applied to the strategy development, is presented in Figure 3. Steps one and two of the model were carried out in parallel, as the needs analysis in step two guided the choice of topics for the development of the guidelines in step one. As part of step two, a previously reported cross-sectional survey [5] was conducted. The implementation strategy developed in step three focused on addressing the determinants of guideline use identified in the survey. Determinants and how they were addressed in our implementation intervention are shown in Figure 4.

Further, an educational element was included in the implementation strategy, to address misconceptions of the EBP concept that emerged from the survey. To guide topic selection, input was also solicited in the survey regarding which guidelines were needed the most. Among the most commonly demanded topics were guidelines for physical therapy treatment of low back pain, neck pain, and subacromial pain. These diagnoses are also referenced in the literature as three of the most common reasons to consult a PT in primary care [45]. In step four, details of the implementation activities were planned and the plan was executed (see below). In step five, data for the evaluation of the implementation were collected with the same questionnaire [46].

### Intervention

The implementation plan carried out in step four of the model comprised a multi-component intervention with the following components:

• Guidelines in printed and electronic formats, with treatment recommendations linked to evidence levels and summarized in a front-page box.

• A 3-hour implementation seminar (core component): The guideline development process, results and recommendations were presented (1.5 hours) together with a learning component on EBP and guidelines (0.5 hour) and integrated interactive group discussions (1 hour).

• A specially developed guideline website, providing easy access to the guidelines, recommended outcome measures, links to medical databases and other EBP resources, and other related materials.

• Links from local intranets to the guideline website, further facilitating access.

• Bi-monthly e-mail reminders with brief “newsletter” style information.

• Patient information leaflets with information and advice on self-care in line with the guideline recommendations, in printed and electronic format.

• E-mail and telephone support by the project manager.

The seminar and group discussion sessions were held on nine different occasions over a 3-month period, with 9–28 PTs attending each session. In the group discussion, the guideline recommendations and current practice were discussed. The sessions were conducted by the first author and a project team co-member, both primary care PTs with postgraduate training in EBP skills. The seminars were held during working hours. Participation was supported and encouraged by senior managers but it was up to each unit manager to decide whether to send all or some of the unit’s PTs. Both the website and the seminar content were tailored to address areas in need of improvement that had been identified earlier [5].

Physical therapists in the control group received no intervention and continued to practice as usual, *i.e.,* according to individual knowledge and experience. They did not receive any of the newly developed physical therapy guidelines, nor any information about the guideline website or the ongoing guideline project. After the follow-up data collection, the guidelines were introduced also to the control group. Publically available physical therapy and multidisciplinary guidelines, searchable on the Internet, were available to both groups during the study.

### Data collection

Data were collected at baseline and follow-up via a web-based, self-report questionnaire covering various aspects of EBP and guidelines [46]. Follow-up data collection took place three to six months after the implementation seminars were carried out in the intervention group, corresponding to one year after baseline data collection.

The primary outcomes were the self-reported awareness of the existence of guidelines, knowledge of how to find guidelines, access to guidelines, and use of guidelines. The first three outcomes were selected because of their role as facilitators of guideline use, and the fourth outcome because of its importance in measuring the extent to which EBP was applied in practice. Awareness, knowledge, and access to guidelines were assessed with 3-point scales “yes”, “partially”, or “no”. Use of guidelines was assessed on a 5-point scale with response alternatives “never or very infrequently”, “infrequently”, “sometimes”, “frequently”, or “very frequently or always”. Secondary outcomes were self-reported attitudes to EBP and guidelines, assessed with 5-point scales ranging from “strongly disagree” to “strongly agree”.

The questionnaire had, in an earlier study, been found to be valid and of acceptable reliability in a primary care physical therapy context [46]. In that study, it was translated, cross-culturally adapted, and further developed from a previously used instrument to assess EBP [3]. Invitation to respond to the questionnaire was distributed via e-mail, containing a link to the questionnaire. Participants responded on-line and the survey software (EPiServer CMS 5, EPiServer AB, Stockholm, Sweden) logged the responses and added them to a results database. At both baseline and follow-up, three reminder notices were e-mailed at 1-week intervals. Staff turnover was analyzed by comparing the e-mail addresses between the two data collection points.

### Ethics

Ethical approval for the study was obtained from the Regional Ethical Review Board of Gothenburg (Reference 780–11). All questionnaires were filled out anonymously and responses could not be traced back to the respondents.

### Data analysis

Sample size was calculated based on the assumption that a relevant difference between groups in the primary outcomes would be 15%. We estimated that we needed 137 PTs in each group to be able to detect a relevant change, with 80% power and a significance level of 5%. Assuming a response rate of approximately 65%, we needed to send the questionnaire to approximately 400 respondents, which corresponded well with the number of PTs employed in primary care in the county council.

Participating practices were analyzed using individual PTs as measurement units. Baseline data were compared between groups for age, gender, years of primary care experience, education level, workplace size, and for primary and secondary outcomes. Differences in proportions between groups were analyzed using Pearson’s χ^2^ test. Before the analysis, the response categories were dichotomized into the two highest and lowest categories. For the primary outcomes, primary analyses of data for all PTs in the intervention group were performed, regardless of whether they had participated in the implementation seminar, the main component of the intervention (“intention-to-treat” analysis). Secondary analysis was restricted to those participants who actually participated in the seminar and responded to the follow-up questionnaire (“per-protocol” analysis).

For the primary outcomes, absolute changes between baseline and follow-up in proportions answering “frequently/almost always” or “yes” were computed. Differences in change between the groups were computed and analyzed using an approximative z-test. A significance level was set at p < 0.05. Analyses were performed using IBM SPSS Statistics, version 20.

## Results

Of the 448 eligible PTs at follow-up, 277 belonged to the intervention group and 171 to the control group. Responses were received from 168 (60.6%) and 88 (51.5%) PTs, respectively. One hundred and eighty-six PTs participated in the implementation seminars, of which 97 (52.2%) responded (Figure 1).

Respondent and workplace characteristics in the two groups were comparable, both at baseline and follow-up, with the exception of workplace size; fewer PTs in the intervention group worked at clinics with 6−10 PTs and more worked at clinics with 11−15 PTs (Table 1). There were no differences in respondent and workplace characteristics within the groups between baseline and follow-up. Staff turnover averaged 10%, meaning that 90% of the study populations in the two surveys were the same.

**Table 1:** Participant demographic and workplace characteristics at baseline and follow-up, by study group

**Characteristic**	**Baseline**		**Follow-up**	
	**Intervention group (n = 171)**	**Control group (n = 100)**	**Intervention group (n = 168)**	**Control group (n = 88)**
Sex (female)	129 (75.4%)	75 (75.0%)	129 (76.8%)	65 (73.9%)
Age (years)				
20−29	20 (11.7%)	13 (13.0%)	29 (17.3%)	20 (22.7%)
30−39	49 (28.6%)	25 (25.0%)	49 (29.2%)	19 (21.6%)
40−49	53 (31.0%)	34 (34.0%)	49 (29.2%)	27 (30.7%)
50−59	41 (24.0%)	23 (23.0%)	33 (19.6%)	16 (18.2%)
60+	8 (4.7%)	5 (5.0%)	8 (4.7%)	6 (6.8%)
Education (years)				
<2.5	8 (4.7%)	9 (9.0%)	10 (6.0%)	6 (6.8%)
2.5	35 (20.5%)	24 (24.0%)	18 (10.7%)	10 (11.4%)
3 (Bachelor’s degree)	116 (67.8%)	65 (65.0%)	126 (75.0%)	70 (79.5%)
≥4 (Postgraduate degree)	12 (7.0%)	2 (2.0%)	14 (8.3%)	2 (2.3%)
Work experience in primary care physical therapy (years)				
< 5	44 (25.7%)	29 (29.0%)	58 (34.5%)	32 (36.4%)
6−10	35 (20.5%)	15 (15.0%)	34 (20.2%)	16 (18.2%)
11−15	28 (16.4%)	16 (16.0%)	23 (13.7%)	10 (11.4%)
16−20	22 (12.9%)	16 (16.0%)	16 (9.5%)	10 (11.4%)
> 20	42 (24.6%)	24 (24.0%)	37 (22.0%)	20 (22.7%)
Specialist	5 (2.9%)	1 (1.0%)	6 (3.6%)	3 (3.4%)
Size of workplace (no. of PTs)				
< 3	15 (8.9%)	13 (13.1%)	11 (6.5%)	8 (9.1%)
3−5	40 (23.7%)	20 (20.2%)	37 (22.0%)	24 (27.3%)
6−10	61 (36.1%)*	56 (56.6%)*	62 (36.9%)*	45 (51.1%)*
11−15	29 (17.1%)*	0 (0.0%)*	37 (22.0%)*	3 (3.4%)*
> 15	24 (14.2%)	10 (10.1%)	21 (12.5%)	8 (9.1%)

Data are numbers (percentages).

*Denotes significant between-group differences.

### Primary outcomes

At follow-up, a significantly higher proportion of PTs in the intervention group (59%) than in the control group (44%) reported being aware of guidelines (p = 0.030), knowing where to find guidelines (40% vs. 16%; p < 0.001), and having easy access to guidelines (26% vs. 7%; p < 0.001) (Table 2). The difference in guideline use was not statistically significant in intention-to-treat analysis (55% vs. 48%; p = 0.081), but was significant in per-protocol analysis, *i.e.,* PTs who participated in the implementation seminar reported frequent use of guidelines to significantly greater extent than those in the control group (63% vs. 48%; p = 0.043).

**Table 2:** Distribution of questionnaire responses for primary outcomes with between-group analyses

**Outcome**	**Baseline**			**Follow-up**		**P value**	**Follow-up**		**P value**
				**Intention-to-treat**			**Per protocol**		
	Intervention group (n = 171)*	Control group (n = 100)*		Intervention group (n = 168)*	Control group (n = 88)*		Intervention group (n = 97)*	Control group (n = 88)*	
*Awareness that guidelines exist*			ns			p = 0.030			p = 0.003
Yes	53 (31.0%)	37 (37.0%)		99 (58.9%)	39 (44.3%)		65 (67.0%)	39 (44.3%)	
Partially	108 (63.2%)	57 (57.0%)		68 (40.5%)	46 (52.3%)		32 (33.0%)	46 (52.3%)	
No	10 (5.8%)	6 (6.0%)		1 (0.6%)	3 (3.4%)		0 (0.0%)	3 (3.4%)	
*Knowledge of where to find guidelines*			ns			p < 0.001			p < 0.001
Yes	25 (14.7%)	11 (11.1%)		67 (39.9%)	14 (15.9%)		45 (46.4%)	14 (15.9%)	
Partially	108 (63.5%)	67 (67.7%)		90 (53.6%)	57 (64.8%)		47 (48.5%)	57 (64.8%)	
No	37 (21.8%)	21 (21.2%)		11 (6.5%)	17 (19.3%)		5 (5.2%)	17 (19.3%)	
*Easy access to guidelines*			ns			p < 0.001			p < 0.001
Yes	14 (8.2%)	11 (11.1%)		43 (25.6%)	6 (6.8%)		31 (32.0%)	6 (6.8%)	
Partially	92 (53.8%)	52 (52.5%)		105 (62.5%)	56 (63.6%)		62 (63.9%)	56 (63.6%)	
No	65 (38.0%)	36 (36.4%)		20 (11.9%)	26 (29.5%)		4 (4.1%)	26 (29.5%)	
*Use of guidelines*			ns			p = 0.081			p = 0.043
Frequently	78 (46.2%)	48 (48.0%)		93 (55.4%)	42 (47.7%)		61 (62.9%)	42 (47.7%)	
Sometimes	70 (41.4%)	41 (41.0%)		69 (41.1%)	37 (42.0%)		33 (34.0%)	37 (42.0%)	
Infrequently	21 (12.4%)	11 (11.0%)		6 (3.6%)	9 (10.2%)		3 (3.1%)	9 (10.2%)	

Data are numbers (percentages); ns, not significant.

*Numbers do not always add up to total n in respective group due to missing data.

Within-group changes in proportions from baseline to follow-up are shown in Table 3. The differences between the groups in this change were also statistically significant for three of the four primary outcomes (Table 3). The proportions of PTs reporting awareness of (p = 0.023), knowledge of (p = 0.007), and easy access to (p < 0.001) guidelines increased more in the intervention group than in the control group. The change in self-reported use of guidelines did not differ significantly (p = 0.297). Differences in proportions were between 9% and 22%.

**Table 3:** Analysis of changes in proportions in questionnaire responses for primary outcomes

**Outcome**	**Intervention group**				**Control group**				**Comparison**		
	**Pre (n = 171)**	**Post (n = 168)**	**Absolute change**	**P value**	**Pre (n = 100)**	**Post (n = 88)**	**Absolute change**	**P value**	**Absolute diff.**	**z**	**P value**
Awareness that guidelines exist (yes)	31.0%	58.9%	27.9%	p < 0.001	37.0%	44.3%	7.3%	ns	**20.6%**	2.281	0.023
Knowledge of where to find guidelines (yes)	14.7%	39.9%	25.2%	p < 0.001	11.1%	15.9%	4.8%	ns	**20.4%**	2.689	0.007
Easy access to guidelines (yes)	8.2%	25.6%	17.4%	p < 0.001	11.1%	6.8%	−4.3%	ns	**21.7%**	3.422	<0.001
Use of guidelines (frequently or almost always)	46.2%	55.4%	9.2%	p = 0.091	48.0%	47.8%	−0.2%	ns	9.4%	1.031	0.302

Absolute diff = difference between groups in change in proportions pre to post intervention.

Significant differences are in bold type.

### Secondary outcomes

Table 4 shows that there were no statistically significant differences in secondary outcomes. The only exception was “EBP helps decision making”, but this difference was small. Per-protocol analyses of secondary outcomes produced similar results (not reported).

**Table 4:** Distribution of questionnaire responses for secondary outcomes with between-group analyses

	**Baseline**		**P value**	**Follow-up**		**P value**
	Intervention (n = 171)*	Control (n = 100)*		Intervention (n = 168)*	Control (n = 88)*	
**Attitudes to EBP**						
*EBP is necessary*			ns			ns
Agree	151 (88.8%)	90 (90.9%)		153 (91.1%)	83 (94.3%)	
Neutral	14 (8.2%)	5 (5.1%)		11 (6.5%)	5 (5.7%)	
Disagree	5 (2.9%)	4 (4.0%)		4 (2.4%)	0 (0.0%)	
*EBP places unreasonable demands*			ns			ns
Agree	37 (22.0%)	21 (21.2%)		41 (24.5%)	33 (37.5%)	
Neutral	41 (24.4%)	18 (18.2%)		24 (14.4%)	14 (15.9%)	
Disagree	90 (53.6%)	60 (60.6%)		102 (61.1%)	41 (46.6%)	
*EBP helps decision making*			ns			p = 0.018
Agree	136 (81.0%)	86 (86.0%)		150 (89.3%)	74 (85.1%)	
Neutral	26 (15.5%)	9 (9.0%)		11 (6.5%)	13 (14.9%)	
Disagree	6 (3.6%)	5 (5.0%)		7 (4.2%)	0 (0.0%)	
*Confident to find research*			ns			ns
Agree	104 (61.9%)	61 (61.9%)		113 (68.1%)	56 (65.1%)	
Neutral	18 (10.7%)	11 (11.1%)		23 (13.8%)	11 (12.8%)	
Disagree	46 (27.4%)	27 (27.3%)		30 (18.1%)	19 (22.1%)	
*Confident to treat patients according to evidence*			ns			ns
Agree	109 (65.3%)	75 (75.0%)		123 (73.2%)	60 (68.2%)	
Neutral	33 (19.8%)	12 (12.0%)		28 (16.7%)	20 (22.7%)	
Disagree	25 (15.0%)	13 (13.0%)		17 (10.1%)	8 (9.1%)	
**Attitudes to guidelines**						
*Important that guidelines exist*			ns			ns
Agree	160 (94.1%)	92 (92.0%)		159 (94.6%)	82 (94.3%)	
Neutral	4 (2.4%)	1 (1.0%)		5 (3.0%)	3 (3.4%)	
Disagree	6 (3.5%)	7 (7.0%)		4 (2.4%)	2 (2.3%)	
*Important to use guidelines*			ns			ns
Agree	164 (95.9%)	97 (97.0%)		160 (95.8%)	84 (95.5%)	
Neutral	6 (3.5%)	2 (2.0%)		6 (3.6%)	3 (3.4%)	
Disagree	1 (0.6%)	1 (1.0%)		1 (0.6%)	1 (1.1%)	
*Can integrate patient pref’s with guidelines*			ns			ns
Agree	121 (71.6%)	63 (63.0%)		138 (82.1%)	68 (78.2%)	
Neutral	42 (24.8%)	32 (32.0%)		29 (17.3%)	18 (20.7%)	
Disagree	6 (3.6%)	5 (5.0%)		1 (0.6%)	1 (1.1%)	

Data are numbers (percentages); ns, not significant.

*Numbers do not always add up to total n in respective group due to missing data.

## Discussion

This study showed that a tailored, multi-component guideline implementation intervention, informed by theories as well as current evidence and structured around an implementation model, can affect the frequency of guideline use, as well as several determinants of guideline use, among PTs in primary care. Attitudes, in general, were not affected. Significant effects were seen in the intervention group in three of the four primary outcomes. The effects were of a moderately large magnitude (20% to 22%) and can be considered relevant. Awareness of the existence of the guideline is a critical prerequisite for the use of guidelines and is logically the first step toward adherence [47]. Awareness and familiarity with the guidelines’ content are likely to affect implementation [19]. Knowing where to find guidelines and having easy access to them are also important prerequisites for the use of guidelines, the latter often considered an organizational or contextual barrier [48].

However, the effect on these facilitators of guideline use did not carry through to the fourth primary outcome. Use of guidelines only increased by an insignificant 9% in the whole intervention group and a significant 17% among those who participated in the seminar, respectively, while remaining unchanged in the control group (Table 2). It is well established that changes in cognitive factors such as knowledge, beliefs, and attitudes do not necessarily translate into behavior change [49,50]. Knowledge is but one of 12 different domains that have been identified to influence implementation of guidelines and EBP [51].

### Comparison to previous studies

Our findings support the conclusions of two systematic reviews [38,39], concluding that PTs’ knowledge and, to some extent, behavior but not attitudes were improved after active multi-component implementation interventions. However, a third systematic review [37] found that multifaceted guideline implementation interventions were no more effective than single intervention strategies across several allied health professions. Two randomized controlled studies (included in the reviews) compared an active implementation intervention versus passive dissemination of physical therapy guidelines for low back pain and whiplash disorders, respectively [40,41]. The active interventions were similar to ours and included interactive training, group discussions, and reminders. The studies found a 12% and a 44%, respectively, higher self-reported guideline adherence rate after the active strategy; a better result than in our study. However, in the study on whiplash guidelines, the effects on adherence were mixed; two of the guideline’s five recommendations were adhered to more by the intervention PTs than by those in the control group, while this was not the case for the other three recommendations.

A recent study of PTs and other musculoskeletal practitioners in the UK, reported a 60% guideline adherence rate after an implementation of guidelines for low back pain via posted information, versus 55% in the no-intervention control group; only a 5% absolute difference [52].

Hence, effect sizes in the few guideline implementation studies that have been published in physical therapy varied between 5% and 44%. In other healthcare areas, effects of implementation interventions are known to be modest. Systematic reviews [53,54] have reported average effects of guideline implementation on performance or process of care between 5% and 10%, consistent with the findings of this study.

The increase in the proportion using guidelines frequently, non-significant in the ITT analysis but significant in per protocol analysis, implies an encouraging trend and the impact is likely to be visible over time as these PT’s patients can be expected to receive guideline-informed, evidence-based treatment. The corresponding effect on *infrequent* guideline use may be even more important. The proportion of PTs who reported infrequent guideline use was reduced from 12% to 4% in the intervention group. The implication of this is that very few PTs practice without consideration of the guidelines after the implementation intervention, which should have a positive impact on the quality of care.

The post-intervention rate of frequent guideline use in our study, 55%, can still be considered quite low, leaving room for future improvement. Changing behavior to start using guidelines more frequently is a process that takes time, and we measured only a few months after the implementation activities. A Dutch survey examining guideline adherence three years after a postal dissemination of physical therapy guidelines for low back pain, reported a corresponding self-reported adherence rate of 61% [55], only slightly higher. In other cross-sectional surveys, varying rates of guideline adherence have been reported among PTs in different countries and settings, ranging from 40% in the United States [2] and 45% in Australia [12], to 75%-86% in Sweden [13,14]. Comparisons are however treacherous, as data were collected and analyzed differently in the studies.

On the other hand, 55% may not be that low. If we add the proportion of PTs who reported using guidelines “sometimes”, the rate increases to a high 96%. “Sometimes” might be a perfectly adequate frequency, *e.g.,* if you don’t see patients with diagnoses for which guidelines exist more often than “sometimes” or if you have integrated guideline recommendations in your clinical decision making to the point that you are not even aware of using them.

### Possible explanations of the results

There are several possible explanations for the modest effect on guideline use. Rogers [56] has suggested that the adoption of new innovations is highly influenced by the innovation characteristics and has described five perceived attributes of the implementation object: relative advantage, compatibility, complexity, trialability, and observability. Grilli and Lomas [57] found that of those, trialability (the degree to which an innovation may be tried on a limited basis) was the most important attribute of guidelines, enhancing adherence, and that complexity was associated with low adherence rates. Compatibility with existing clinical practice has also been shown to enhance adherence [58]. In our project, trialability and complexity were addressed already in the development of the guidelines. Because the guideline recommendations did not differ much from existing practice, compatibility was also achieved. However, this very fact could maybe explain the modest effect on the use of guidelines; since they did not introduce any new revolutionary methods but rather confirmed existing practice, using them frequently might not be perceived as necessary.

Generally, it has been suggested that the modest effect of most implementation interventions could be due to the complexity of implementation research [59]. The primary health care context involves many stakeholders at multiple levels, creating significant conceptual and methodological challenges such as developing effective study designs and selecting appropriate outcomes. Furthermore, to achieve a practice change, managerial and organizational support for an implementation project is crucial.

The fact that the per-protocol analysis yielded significant effect on guideline use where the intention-to-treat analysis did not, could either suggest that participating PTs did not share the content of the implementation seminar sufficiently with non-attending colleagues, or that there was a diffusion effect between the intervention and control groups. This finding indicates the importance of implementation seminar attendance, which therefore should be more strongly encouraged or mandated by managers.

Naturally, the positive effect on awareness, knowledge, and access were likely related to the components of the intervention that addressed these outcomes, and the specifically created website was a main contributor. Verbal information at the implementation seminars, supplemented by well-structured guideline information and supporting documents on the website as well as regular e-mail reminders, is likely to have contributed to increased awareness, knowledge and perceived easy access.

Attitudes to EBP and guidelines were for the most part not affected. It would have been difficult to demonstrate statistical significance within this study because participants in both groups were already very positive at baseline; hence there was little room for improvement.

The rather high baseline levels of awareness and use of guidelines (in both groups), also likely contributors to the modest effects on guideline use, could be explained by how the survey questions were phrased and the availability of other guidelines. PTs with good search skills and an interest in searching for guidelines could have searched and found guidelines on their own initiative. The questions were phrased in a rather general way: “are you aware of evidence-based clinical practice guidelines relevant for your practice?” and “how often do you use evidence-based clinical practice guidelines?”. We didn’t ask about the study specific guidelines since they were to be developed. At the time of the follow-up, PTs in the intervention group most likely interpreted the questions as referring to the recently developed and introduced guidelines, whereas the control group most likely interpreted the questions in a general way. Thus, the high baseline rates of guideline use imply that other guidelines than those introduced in the intervention, were used in both groups.

### Implementation strategy

The use of a structured model to guide the implementation project was perceived as useful by the project team. Because the content and recommendations of the guidelines did not differ much from existing practice, complex theories were not considered necessary. However, because the model by Grol et al. [17] is rather pragmatic and integrates several theories in a non-explicit way, it may be less suitable for gaining a deeper understanding of how change was achieved. Linking intervention components, or behavior change techniques, to specific theories of behavior change, could be a more effective strategy to reach this goal [60].

Tailoring implementation components to identified determinants was also perceived as a useful and logical strategy. The core component of the intervention, the implementation seminar, together with the website, addressed most of the identified determinants. The inclusion of interactive group discussions has been suggested as one of the possible tailoring mechanisms that could enhance the impact of educational meetings in implementation [61]. Few studies have been performed that use a tailored strategy to implement change in physical therapy practice. Stevens and Beurskens developed and evaluated a tailored strategy to implement measurement instruments in a Dutch physical therapy context [62]. They concluded, based on interviews with a small group of PTs, that the strategy was applicable to physical therapy practice and effective in improving awareness, knowledge, attitude and use of measurement instruments.

As illustrated in Figure 3, we linked the components of our intervention to the identified determinants, as recommended in the literature [28]. In addition to identifying barriers and facilitators in our population via the baseline data collection, we supplemented these findings with a literature search. Using more than one method to identify determinants is recommended [63]. The largest barrier identified, lack of time, was addressed indirectly by attempting to reduce several of the other barriers, such as poor awareness, availability of and access to guidelines, *i.e.*, decreasing the time required to search for evidence. It was also addressed in the guideline development process, as the guidelines were developed with this barrier in mind. They were designed to be brief and concise. The characteristics of the guidelines were carefully considered as they are likely to affect actual use. Guidelines that are easy to understand, can easily be tried out, and do not require specific resources have a greater chance of implementation [19]. Lack of time is however mainly an organizational or contextual barrier that was not possible to address directly in this intervention.

While social cognitive determinants such as the ones addressed in our study have been shown also in earlier research to influence clinical behavior [64], in particular the use of research evidence [65], a less explored factor is the role of habits in clinical behavior. In a fairly stable context such as the one in this study, clinical practice can be assumed to be habitual and habits, *i.e.,* repeated behaviors, can in stable contexts be difficult to change [50]. Especially in a situation where the guideline recommendations did not differ much from existing practice, many PTs may not have felt a necessity to change their habits and start using guidelines more frequently.

Other possible strategies would have been to use audit and feedback or local opinion leaders. However, according to Grimshaw et al’s recent review on various implementation strategies [21], audit and feedback seem to be less effective than educational strategies. The one strategy that stands out, with a median effect size of 12% versus 4-6% for other strategies, is the use of local opinion leaders. This was not known at the time of our strategy development, nor were there any obvious candidates in our study setting. This strategy would require the availability of credible and influential persons, as well as an intact social professional network [21]. With those conditions satisfied, it would be a recommended strategy in future implementation endeavours.

### Generalizability

The study population is likely to be broadly similar to the national population of primary care PTs in Sweden. Although the study was conducted in only one county council/geographic region, this particular county council is the second largest in Sweden. Thus, we believe that our results can be generalized to PTs working in primary care in other county councils in Sweden and possibly also to other countries, particularly those in which, like Sweden, PTs have a large degree of autonomy.

### Methodological considerations

The study has several limitations. Only process outcomes at practitioner level were measured and we do not know whether the intervention had, or will have, an impact on patient outcomes. We only measured self-reported behavior, which entails a risk of overestimation and social desirability bias, and limits our ability to draw strong conclusions concerning actual behavior [66].

To minimize social desirability bias, the questionnaires were anonymous and it was therefore not possible to track individual responses and changes between the two test occasions. Statistically, only comparisons between independent groups were possible. However, staff turnover was rather low during the study period, with an average of 90% of PTs remaining the same in the respective groups from baseline to follow-up measurements. The study population can therefore be considered rather stable.

As with all non-randomized studies, it cannot be fully ascertained that the observed changes can be attributed to the intervention. Randomization was in this project not feasible, as a likely diffusion effect between participants would have made data interpretation difficult. Instead, the second strongest design, a controlled before–after design with a comparable control group, was used [59]. Data were collected in both populations contemporaneously using the same method at baseline and follow-up. A ‘between group’ analysis comparing performance in the study and control groups was performed, and the observed differences could be assumed to be due to the intervention [67]. Both groups were similar at baseline and could be expected to experience secular trends or changes in the same extent, increasing the confidence with which the observed changes can be attributed to the intervention [67].

Communication and interaction between PTs in the intervention and control group on individual or group levels, *e.g.,* meetings, conferences, were not unlikely, causing a risk for contamination between the two groups and reducing the apparent effect of the intervention.

The short duration between implementation and follow-up evaluation is likely to have contributed to the modest effect on guideline use. The introduction of complex workplace change, especially as concerns EBP, takes considerable time [68]. The actual use of, or adherence to, a guideline has been described as the last in a sequence of cognitive and behavioral steps that begin with awareness [47], and to expect a high impact on guideline use so soon after the intervention is probably not realistic. A longer follow-up period would have been useful but was not possible due to the guidelines and website being made available also to the control group after the follow-up survey was conducted.

Another limitation, possibly contributing to the modest effect on guideline use, could be that the education component of the implementation intervention comprised only one session. Two systematic reviews have shown that educational meetings can improve both professional practice and patient outcomes and that sessions with interactive components may have greater impact [23,69]. However, the effectiveness of various educational strategies is not conclusive and factors such as time and intensity have been proposed to influence outcomes [18]. One single session may not be enough, although we took effort to make it as interactive as possible. Interactive educational strategies have consistently been reported as more effective than traditional, didactic education and engaging the clinician is recommended [18]. Additional educational sessions might improve the effect of the implementation, but this would need to be investigated.

The intervention focused on the individual level. Mid-level managers have a potentially pivotal role as implementation agents and their proactivity and commitment have been suggested to influence implementation effectiveness [70]. A multi-level approach and particularly targeting also unit managers would likely have increased the impact of our implementation intervention.

Although the questionnaire used was previously validated in a similar population of primary care PTs and found to have acceptable reliability, there are some interpretation issues. The response categories (*e.g.,* what is “frequently”?) may have been interpreted differently by the respondents. In the data analysis, the categorization/dichotomization of the responses is not obvious, and may have been performed differently in different studies, rendering comparison difficult.

### Implications for research

Measuring the effect of implementation interventions as well as guideline use is very complex, but very important. To build upon the results of this study, further research is recommended that evaluates an implementation strategy that comprises more than one educational session, and that is designed to enable a longer follow-up period, *e.g.,* two years. Using a more rigorous design, *i.e.,* a randomized trial, is paramount to reduce risk of bias and be able to draw stronger conclusions. In view of the modest effect on guideline use and the considerable resources required for an active, multi-component intervention such as the one in this study, a comparison between different implementation strategies with various components could provide valuable knowledge. A strategy that needs to be evaluated in the physical therapy context would be the use of local opinion leaders. Linking intervention components, or behavior change techniques, to specific theories of behavior change could facilitate a greater understanding of what works and what doesn’t. Data should be collected so that individual changes from baseline to follow-up can be analyzed. It is also very important to measure the effect on patient outcomes as well as the cost-effectiveness of guideline implementation strategies. Furthermore, quantitative evaluation studies need to be accompanied by qualitative studies to achieve increased understanding of effect mechanisms of implementation interventions and practice behavior change. Lastly, the patient’s voice is seldom heard in implementation research and patient views and preferences should be included in an evaluation of the barriers and facilitators for implementation, development of the implementation intervention, and in choosing relevant outcomes.

### Implications for practice

The implementation of the guidelines can be expected to have an impact on physical therapy practice in the target population in several phases. In a first phase, the short term effects of the implementation – improved awareness of guidelines, knowledge about where to find them, and ability to access them easily through the dedicated website – will set the stage for a second phase, increased use of the guidelines thereby facilitating the application of EBP. In a third, long-term phase, patients could be expected to benefit from this, as they will be treated with methods known to be the most effective according to current evidence. This could, however, be expected to take quite some time. The effect of a guideline implementation, such as the one performed in this study, might be enhanced by conducting additional educational sessions. On the other hand, it might also be relevant, and more cost-effective, to consider a scaled-down educational strategy, such as training “ambassadors” that can train PTs at their workplace instead of having them all come to larger, out-of-office seminars. Organizational barriers could also be addressed to a larger extent, *e.g.*, by securing active support from senior management and arranging for further discussions of the guidelines at the workplace. Decision makers need to carefully consider whether the benefits of implementation efforts are important enough to outweigh the costs involved.

## Conclusions

In conclusion, this study demonstrates that a tailored, multi-component intervention for the implementation of clinical practice guidelines, informed by theory as well as current evidence, had a positive effect on self-reported awareness of the existence of guidelines, knowledge of how to find guidelines, and access to guidelines in PTs in primary care in western Sweden. While these factors are important prerequisites to and determinants of guideline use, the self-reported use of guidelines was not affected to the same extent. The results support previous findings on physical therapy guideline implementation strategies that active, multi-component strategies are effective but that the effects are modest.

## Competing interests

The authors declare that they have no competing interests. The first author was project manager for the guideline development and implementation projects. All authors are responsible for the content and writing of the paper.

## Authors’ contributions

SB contributed to the design, data acquisition and analysis, and drafted the manuscript. ML participated in design, data analysis and interpretation, and drafting of the manuscript. KJ, PN and BÖ contributed to data analysis and interpretation, and drafting of the manuscript. RE, MFO, LN and MvT contributed to the design and critically revised the manuscript. All authors read and approved the final manuscript.

## Pre-publication history

The pre-publication history for this paper can be accessed here:

http://www.biomedcentral.com/1472-6963/14/105/prepub
